# Incipient Melanonychia: Benign Finding or Occult Malignancy? A Case Report of Subungual Melanoma

**DOI:** 10.7759/cureus.34292

**Published:** 2023-01-27

**Authors:** Alejandro J Quiroz Alfaro, Juliana Greiffenstein, Andrés Felipe Herrera Ortiz, Catalina A Dussan Tovar, Sara Saldarriaga Santamaría, Jaqueline Cifuentes Burbano, Nataly García, Maria C Rodríguez Díaz, Susana M Sierra Molina, Guillermo Jiménez Calfat

**Affiliations:** 1 Medicine and Health Sciences, Universidad Colegio Mayor de Nuestra Señora del Rosario, Bogotá D.C., COL; 2 Facultad de Medicina, Universidad Pontificia Bolivariana, Medellín, COL; 3 Radiology, Fundación Santa Fe de Bogotá, Bogotá D.C., COL; 4 Radiology, Universidad El Bosque, Bogotá D.C., COL; 5 Epidemiology, Universidad de La Sabana, Chía, COL; 6 Dermatology, Universidad CES, Medellín, COL; 7 Dermatopathology, Universidad CES, Medellín, COL; 8 Medicine, Universidad San Martín, Medellín, COL; 9 Medicine, Universidad el Bosque, Bogotá D.C, COL; 10 Medicine, Universidad de los Andes, Bogotá D.C., COL; 11 Surgical Oncology, Universidad CES, Medellín, COL

**Keywords:** internal medicine, nail matrix tumor, skin neoplasm, dermatology, mohs surgery, histopathologic diagnosis, immunohistochemistry, onychoscopy, melanonychia, subungual melanoma

## Abstract

Subungual melanomas are rare neoplasms that tend to debut as longitudinal melanonychia. They are primarily found in patients over 60 years of age and are usually diagnosed late, representing a diagnostic challenge. We present a case report of a 59-year-old female Hispanic patient who initially presented with melanonychia and was eventually diagnosed with subungual melanoma in situ. She was surgically treated and, after three months, remained healthy. Relevant risk factors, clinical and onychoscopic findings, diagnostic criteria, and treatment options are also discussed. Since many benign entities present similarly, high clinical suspicion is critical for diagnosing this entity.

## Introduction

Melanonychia (ML) is defined as a black, brown, or gray pigmentation of the nail plate. It is usually caused by the activation and proliferation of melanocytes from the distal nail matrix [1]; however, it can also be caused by fungal infections of the nail, subungual hematomas, exogenous pigments, systemic disorders, and melanoma [2].

ML has a variable prevalence between 0.8% and 23% worldwide, and it is more commonly found in Asian and African American populations [3]. Depending on its presentation, it can be classified as transversal, longitudinal, or diffuse; longitudinal ML is the most common pattern [4].

Subungual melanoma (SM) is a rare neoplasm accounting for 0.31%-2.8% of all cutaneous melanoma cases. It is primarily found in patients between 60 and 70 years of age and is commonly diagnosed late, with a poor prognosis [5,6]. Therefore, clinicians should be familiar with the most common risk factors as well as clinical and onychoscopic findings of SM. In this article, we present a case of a patient with incipient longitudinal ML who was diagnosed with SM.

## Case presentation

A 59-year-old Hispanic female patient presented with an asymptomatic pigmented lesion on the fifth toe of her left foot. She could not recall when she had noticed it for the first time. She did not report any history of trauma, relevant family history, or using any ointments or medications.

**Figure 1:**
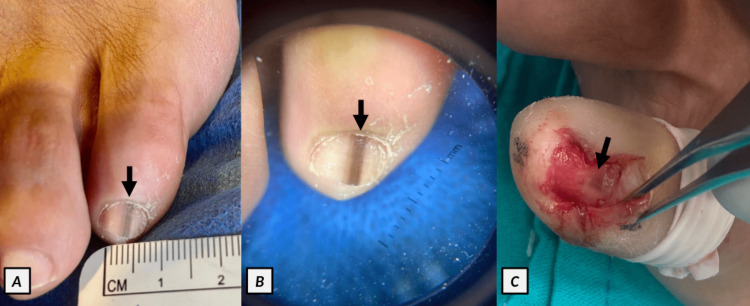
(A) Longitudinal melanonychia on the fifth toe of the left foot (black arrow). (B) Onychoscopy of the toe showing regular, parallel, brown-pigmented lines forming a band along the nail plate (black arrow). (C) Intraoperative image showing the presence of pigment on the distal nail matrix (black arrow).

On physical examination, a longitudinal, brown-colored, pigmented band of approximately 2 mm breadth was seen on the nail plate of the toe (Figure 1, Panel A).

Onychoscopy showed a homogeneous brown pigmentation band with parallel lines, symmetrical breadth, and regular borders on the nail plate. No periungual brownish pigmentation of the cuticle (micro-Hutchinson’s sign) was observed (Figure 1, Panel B). No other skin lesions were present, and the rest of the physical examination was unremarkable. The skin lesion was initially considered benign owing to the above-mentioned characteristics, and the patient was conservatively treated and followed up.

**Figure 2:**
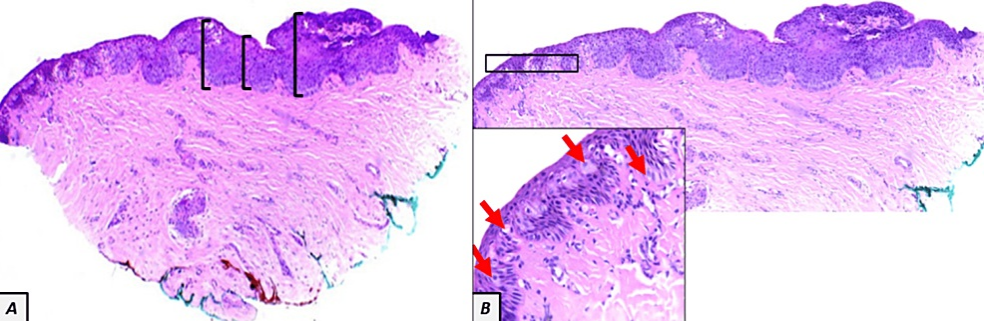
(A) Hematoxylin and eosin stain, x4. Microscopy of the nail matrix tissue specimen shows epithelial acanthosis (black brackets) and pagetoid upward migration of individual epidermal cells with clear cytoplasm and groups of these cells in a spindle shape. (B) Hematoxylin and eosin stain, x10 and x40, respectively. Microscopy of the nail matrix tissue specimen with greater magnification shows the individual epidermal cells with clear cytoplasm (red arrows).

Three months later, the lesion started to darken and become irregular; thus, a nail matrix biopsy was done. After completely removing the nail plate, the presence of pigment on the distal nail matrix was evident; therefore, an excisional biopsy of the pigmented lesion was performed (Figure 1, Panel C). The histopathology of the tissue specimen reported a nail matrix with epithelial acanthosis and pagetoid upward migration of epidermal cells with clear cytoplasm (Figure 2, Panels A and B).

**Figure 3:**
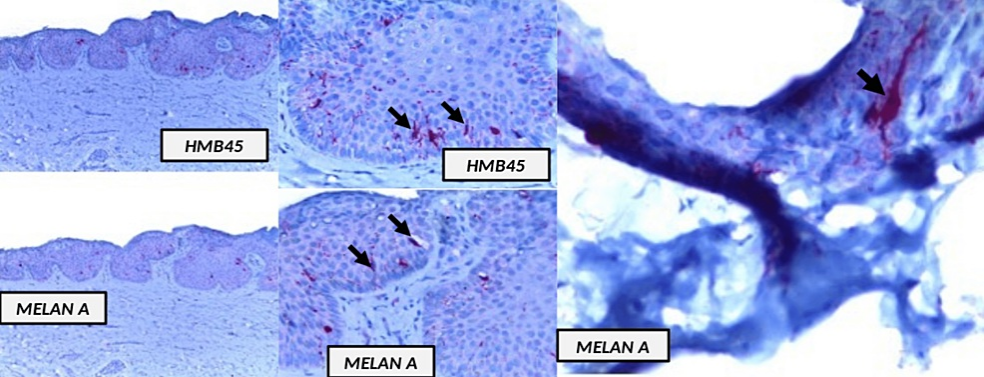
Immunohistochemistry showing Melan-A-positive cells and HMB45-positive cells (black arrows)

Since the histological findings were subtle, immunohistochemistry was done. This showed Melan-A-positive cells and HMB45-positive cells (black arrows) (Figure 3).

Confirming the diagnosis of melanoma in situ

After discussing different treatment options, risks, and benefits, the patient was scheduled for a Mohs surgery. A wide excision with 10 mm margins was performed, achieving complete histological clearance. The patient did not show any signs of recurrence at a three-month follow-up.

## Discussion

Misdiagnosing SM leads to an 18-month median delay in diagnosis and lower estimated five-year survival rates. It is also associated with more advanced staging at the time of diagnosis [7]. The etiology of SM is unclear; however, genetics as well as environmental and repetitive trauma have been recognized as risk factors. Unlike cutaneous melanoma, there is no clear association between sunlight exposure and SM [5,6]. Nail dystrophy, irregular borders, micro-Hutchinson’s sign, and the sudden appearance of a melanocytic lesion are onychoscopic findings that suggest malignancy [2]. Although a biopsy is required to confirm the diagnosis of SM, Levit et al. [8] devised the ABC for the clinical identification of SM (Table 1).

**Table 1:** The ABC for subungual melanoma Source: This table is adapted from Levit et al. [8].

A	Age	Fifth to seventh decades of life, especially in African American, Asian, and Native American patients
B	Brown to black Band Breadth ≥ 3 mm	A band with a breadth of ≥3mm and variegated borders
C	Change	Nail band changes or lack of nail morphology changes despite treatment
D	Digit most commonly involved	Thumb > Hallux > Index finger > Single digit > Multiple digits
E	Extension	The extension of pigment involving the proximal or lateral nail folds (Hutchinson's sign) or the free edge of the nail plate
F	Family	Family or personal history of melanoma or dysplastic nevus

This method can improve the early detection and survival rates of SM. As our patient did not initially have any of the associated risk factors or any of the onychoscopic findings suggesting malignancy, and only her age (she was in the sixth decade of life) was present in the ABC, she was followed up until the lesion changes were noticed during the three-month follow-up onychoscopy.

There has yet to be a consensus regarding the frequency of follow-ups for ML. Some authors recommend following up every six months [5]. However, it is relevant to mention that in our case, changes suggesting malignancy were seen during the three-month follow-up. Therefore, clinicians should be aware that changes may present earlier. Another reason to follow up on ML that does not exhibit findings compatible with malignancy instead of performing the matrix biopsy right away is that permanent nail dystrophy can be a complication of any technique used to biopsy the nail matrix [9]. In our experience, this is an alarming sequela for many patients.

Incipient ML in the fifth decade of life is concerning and can be an indication for biopsy. On the other hand, the fifth toe is not a particularly common location for SM as it is more commonly found in the thumb, hallux, or index finger [6]. Moreover, it was unclear when the lesion appeared in our case as the patient did not remember when she had first noticed it. Thus, following up on a trimestral basis was considered the best option.

Histopathology is the gold standard for the diagnosis of SM. According to Darmawan et al. [10], microscopic findings suggesting SM in situ include multinucleation, melanocytes with hyperchromatic or irregular nuclei, prominent cellular atypia in the irregularly distributed pagetoid melanocytes, irregular melanocytic nests, and predominantly single-cell growth patterns. The histopathology of our case, accompanied by the lack of other signs or symptoms and the excision margins free of cancerous cells, is compatible with SM in situ.

On many occasions, identifying melanocytes in the nail matrix is difficult; consequently, immunohistochemistry can be used for confirmation. In our case, we utilized the markers HMB45 (a monoclonal antibody that binds to melanosome-specific glycoprotein gp100) and Melan A (a protein product of the *MART-1* gene found in the cytoplasm of mature melanocytes), with a sensitivity of 75% and a specificity of 96.9% for HMB45 and a sensitivity of ≥75% and a specificity of 97% for Melan A [6].

Another specific marker used to diagnose SM is the PReferentially expressed Antigen in MElanoma (PRAME). PRAME is a melanoma-associated antigen expressed in cutaneous melanomas and other malignant neoplasms; it has limited expression in benign tumors like benign nevi and some normal tissues (testis, adrenals, ovary, and placenta) [11]. PRAME helps differentiate benign from malignant nail melanocytic lesions with a sensitivity of up to 77.8% and a specificity of 97.6% for SM [12]. Usually, when more than 75% of tumor cells are positive for PRAME, this finding supports the diagnosis of melanoma [11]. However, when the immunohistochemistry was done on the tissue specimen from our patient, this marker was not available at our institution.

For melanoma in situ, treatment options are wide local excision or Mohs micrographic surgery. The purpose is to have margins of 5-10 mm free of cancerous cells [13]; for this reason, our patient underwent 10 mm excision margins, and the treatment was considered successful.

Finally, it is relevant to mention that many primarily benign entities such as melanocytic nevi and lentigines can present as longitudinal melanonychia (LM), similar to how up to 75% of SM present during the initial stages [9]. Hence, it is paramount for clinicians to recognize the risk factors as well as clinical and onychoscopic findings of SM for early diagnosis, treatment, and positive impact on the patients' survival.

## Conclusions

Subungual melanomas are rare neoplasms that tend to present similarly to benign ungueal conditions and tend to be diagnosed late, negatively impacting the patients’ survival. Hence, a high degree of clinical suspicion is essential for early diagnosis and treatment. Although some authors recommend regular follow-ups on ML every six months, changes suggesting malignancy may appear earlier, like in our case. Clinicians must be aware of the most common risk factors as well as clinical and onychoscopic findings of SM as permanent nail dystrophy can be an alarming sequela after performing a nail matrix biopsy.

## References

[1] Piraccini BM, Dika E, Fanti PA (2015). Tips for diagnosis and treatment of nail pigmentation with practical algorithm. Dermatol Clin.

[2] André J, Lateur N (2006). Pigmented nail disorders. Dermatol Clin.

[3] Singal A, Bisherwal K (2020). Melanonychia: etiology, diagnosis, and treatment. Indian Dermatol Online J.

[4] Alessandrini A, Dika E, Starace M, Chessa MA, Piraccini BM (2021). Diagnosis of melanonychia. Dermatol Clin.

[5] Koga H, Saida T, Uhara H (2011). Key point in dermoscopic differentiation between early nail apparatus melanoma and benign longitudinal melanonychia. J Dermatol.

[6] Nevares-Pomales OW, Sarriera-Lazaro CJ, Barrera-Llaurador J, Santiago-Vazquez M, Lugo-Fagundo N, Sanchez JE, Sanchez JL (2018). Pigmented Lesions of the Nail Unit. Am J Dermatopathol.

[7] Metzger S, Ellwanger U, Stroebel W, Schiebel U, Rassner G, Fierlbeck G (1998). Extent and consequences of physician delay in the diagnosis of acral melanoma. Melanoma Res.

[8] Levit EK, Kagen MH, Scher RK, Grossman M, Altman E (2000). The ABC rule for clinical detection of subungual melanoma. J Am Acad Dermatol.

[9] Ruben BS (2010). Pigmented lesions of the nail unit: clinical and histopathologic features. Semin Cutan Med Surg.

[10] Darmawan CC, Ohn J, Mun JH (2022). Diagnosis and treatment of nail melanoma: a review of the clinicopathologic, dermoscopic, and genetic characteristics. J Eur Acad Dermatol Venereol.

[11] Lezcano C, Jungbluth AA, Busam KJ (2021). PRAME immunohistochemistry as an ancillary test for the assessment of melanocytic lesions. Surg Pathol Clin.

[12] Parra O, Linos K, Li Z, Yan S (2022). PRAME expression in melanocytic lesions of the nail. J Cutan Pathol.

[13] Fernández-Horcajuelo J, Espinosa-Lara P, Simón-Lázaro M (2021). [Ungueal melanoma in situ]. Semergen.

